# Listening to Rhythmic Music Reduces Connectivity within the Basal Ganglia and the Reward System

**DOI:** 10.3389/fnins.2017.00153

**Published:** 2017-03-28

**Authors:** Hans P. Brodal, Berge Osnes, Karsten Specht

**Affiliations:** ^1^Department of Biological and Medical Psychology, University of BergenBergen, Norway; ^2^Bjørgvin District Psychiatric Centre, Haukeland University HospitalBergen, Norway; ^3^Department of Clinical Engineering, Haukeland University HospitalBergen, Norway

**Keywords:** music, rhythm, basal ganglia, reward system, ventral striatum, nucleus accumbens, fMRI, dynamic causal modeling

## Abstract

Music can trigger emotional responses in a more direct way than any other stimulus. In particular, music-evoked pleasure involves brain networks that are part of the reward system. Furthermore, rhythmic music stimulates the basal ganglia and may trigger involuntary movements to the beat. In the present study, we created a continuously playing rhythmic, dance floor-like composition where the ambient noise from the MR scanner was incorporated as an additional instrument of rhythm. By treating this continuous stimulation paradigm as a variant of resting-state, the data was analyzed with stochastic dynamic causal modeling (sDCM), which was used for exploring functional dependencies and interactions between core areas of auditory perception, rhythm processing, and reward processing. The sDCM model was a fully connected model with the following areas: auditory cortex, putamen/pallidum, and ventral striatum/nucleus accumbens of both hemispheres. The resulting estimated parameters were compared to ordinary resting-state data, without an additional continuous stimulation. Besides reduced connectivity within the basal ganglia, the results indicated a reduced functional connectivity of the reward system, namely the right ventral striatum/nucleus accumbens from and to the basal ganglia and auditory network while listening to rhythmic music. In addition, the right ventral striatum/nucleus accumbens demonstrated also a change in its hemodynamic parameter, reflecting an increased level of activation. These converging results may indicate that the dopaminergic reward system reduces its functional connectivity and relinquishing its constraints on other areas when we listen to rhythmic music.

## Introduction

Playing and enjoying music is an universal phenomenon and can be found in all known present and past cultures (Gray et al., 2001; Zatorre and Krumhansl, 2002; Fritz et al., 2009); and emotional responses to music are an experience that almost everyone has felt. Further, in all cultures, across all ages, and without the requirement of musical expertise, people easily dance and synchronize their body movements to rhythmic music (Phillips-Silver and Trainor, 2005). Consequently, the recent two decades have seen an increasing interest in mapping the neuroanatomical correlates of music processing for understanding the underlying mechanism that make music such a unique stimulus in human culture and—although controversially discussed—human evolution (Levitin and Tirovolas, 2009). It turns out that the processing of music does not only activate the auditory system but a complex and distributed network, covering cortical and sub-cortical areas (Peretz and Zatorre, 2005; Zatorre and McGill, 2005; Koelsch, 2011, 2014; Zatorre, 2015). Two aspects have been in the closest focus over the recent years: Processing of rhythm and emotions evoked by music.

It is a daily experience that listening to predominantly rhythmic music often spontaneously triggers toe tapping, foot tapping, or head nodding, synchronous with the beat, and is perceived as pleasurable. It is an almost automatic and often involuntary process that does not require cognitive awareness in order to perform rhythmical movements in synchronization with the perceived beat (Phillips-Silver and Trainor, 2005; Trost et al., 2014). It also requires no musical skills or formal musical training, and is mostly and easily triggered by auditory stimulation. It has been shown that rhythm processing, i.e., both the production of rhythmic movements and the perception of rhythmic sounds, activates the basal ganglia and here the putamen in particular, the supplementary motor area (SMA), pre-SMA, and cerebellum (Grahn and Brett, 2007; Zatorre et al., 2007; Chen et al., 2008a,b; Grahn and Rowe, 2009); but also other substructures have been identified as structures that encode musical meter, like the caudate nucleus (Trost et al., 2014). Moreover, studies have also shown that the perception of rhythms with a preferred tempo increases activity in the premotor cortex (Kornysheva et al., 2010).

Besides rhythm, there is mounting evidence that listening to music induces emotional experiences and could interact with the affect systems. Neuroanatomically, these processes are tightly linked not only to the amygdala, but to a network that is involved in expecting and experiencing reward (Levitin and Tirovolas, 2009; Chanda and Levitin, 2013; Koelsch, 2014; Salimpoor et al., 2015). In particular the ventral striatum, comprising the caudate nucleus and the nearby nucleus accumbens together with the ventral tegmental area, the ventral pallidum and other, mainly frontal areas have been repeatedly related to music listening (Blood and Zatorre, 2001; Levitin and Tirovolas, 2009; Chanda and Levitin, 2013; Salimpoor et al., 2013, 2015; Zatorre and Salimpoor, 2013; Koelsch, 2014; Zatorre, 2015). Interestingly, Salimpoor et al. (2011) demonstrated an anatomically distinct increased release of dopamine, depending on whether an emotional response to music was anticipated or experienced. While the dorsal striatum demonstrated an elevated release of dopamine only during the anticipation of an emotional response to the music, the nucleus accumbens demonstrated this during the experience of an emotional response (Salimpoor et al., 2011). Further, the activity of the nucleus accumbens also served as a predictor of the amount one would spend for the music (Salimpoor et al., 2013).

The above-described aspects of emotional responses to music and synchronized movement to rhythmic auditory stimulation are interweaved, particularly in musical groove. Groove is often defined as the musical quality that induces movement and pleasure (Madison, 2006; Madison et al., 2011; Janata et al., 2012; Witek et al., 2014). Further, a recent transcranial-magnetic stimulation study demonstrated that musical groove is able to modulate excitability within the motor system, with stronger motor-evoked potentials to high-groove music (Stupacher et al., 2013). From a musicology perspective, there is strong evidence that the experience of groove is mainly triggered by beat density and syncopation, but also a moderate, i.e., not too simple and not too complex, rhythmic complexity (Stupacher et al., 2013; Witek et al., 2014, 2015). An example for this type of music is the genre of electronic dance music (Panteli et al., 2016), to which also the stimulation of the present study would belong.

Based on the above-described studies, it is evident that rhythmic music, like electronic dance music, triggers responses in different parts of the basal ganglia loop. In particular, the ventral striatum and the putamen appear as key areas that respond to the rhythm of the music and may generate an emotional response. Therefore, it was hypothesized that rhythmic music will affect the connectivity within a network comprising the auditory cortex, putamen/pallidum, and ventral striatum/nucleus accumbens.

This hypothesis was tested using a continuous stimulation paradigm and a resting-state like analysis approach by using stochastic dynamic causal modeling (sDCM). This procedure does not test activation strength *per se*, since there is no reference condition for a contrast, but examines fluctuations of the BOLD signal and functional dependencies between regions. DCM, in general, allows the specification of models with up to eight anatomically distinct regions that are either directly or indirectly connected to each other. Here, the model space was restricted to a plausible model of six areas, representing the sensory input, the rhythm processing in the basal ganglia, and the reward system.

## Methods

### Participants

The participants were recruited from the student population at the University of Bergen. In total, 26 right-handed, healthy adults (music group: eight female, five male, mean age 30.8 ± 8.4, control group: six female, seven male, mean age 22.8 ± 3.7,) participated in this fMRI study. All participants gave written informed consent in accordance with the Declaration of Helsinki and institutional guidelines, and an approval of fMRI studies in healthy subjects was obtained from the regional ethics committee for western Norway (REK-Vest). Half of the subjects listened to the music during the scanning, while the other 13 subjects served as control subjects with an ordinary resting-state fMRI condition without any additional auditory stimulation. Both groups got the instruction to lie still with eyes open. Participants were compensated for their effort with 200 NOK. Exclusion criteria were neurological or psychiatric disorders, claustrophobia, any surgery of the brain, eyes, or head, pregnancy, implants, braces, large tattoos, and non-removable piercing. All participants got an emergency button and were informed that they could withdraw from the study at any point. Participants were recruited mainly through announcements at the University of Bergen and the Haukeland University Hospital.

### Stimuli

To overcome limitations from earlier studies, where often short or chunked pieces of music have been used, the presented study used a new continuous-stimulation design with an experimentally controlled composition, which was synchronized with the sounds generated by the MR scanner. The music was created as electronic dance music, out of 12 samples of different instruments, like dance drums, bass, kick snare, guitar, etc., mixed together with Adobe Audition 2.0 (www.adobe.com) to a 10.16 min-long sequence with 120 beats per minute (see Supplementary Material).

This piece of music was composed with repeated periods of 20 s duration where all the instruments were playing, alternating with periods where only a few instruments were playing. However, the overall rhythm was always present during the entire sequence of 10.16 min. During image acquisition, the TR was set to 2 s, which included a 0.5 s silent gap. By doing so, the MR scanner was synchronized with the rhythm of the music and thereby was acting as “additional” instrument and was not perceived as an unrelated and unsynchronized auditory stimulation. During the scanning, the subjects of the music group were asked to relax and to listen to the music, and were also asked not to move during the scanning. Participants were interviewed after the examination and all except one reported to have experienced the music as pleasant and relaxing. The control subjects were just asked to relax. Although the control subject were examined with the same sequence, i.e., with brief silent gaps of 500 ms, they did not report to have perceived the scanner noise as any kind of music, and they were only debriefed afterwards that they served as control participants in a study that focuses on rhythm perception.

The fMRI study was performed on a 3T GE Signa Excite scanner, and the axial slices for the functional imaging were positioned parallel to the AC–PC line with reference to a high-resolution anatomical image of the entire brain volume, which was obtained using a T_1_-weighted gradient echo pulse sequence. The functional images were acquired using an echo-planar imaging (EPI) sequence with 311 volumes, each containing 24 axial slices (64 × 64 matrix, 3.4 × 3.4 × 5.5 mm^3^ voxel size, TE 30 ms) that covered the cerebrum and most of the cerebellum. This low spatial resolution was selected to gain a short acquisition time of TA 1.5 s. Together with a silent gap of 0.5 s this resulted in an effective TR time of 2 s. The stimuli were presented through MR compatible headphones with insulating materials that also compensated for the ambient scanner noise by 24 dB.

### Data analysis

The DICOM data were converted into 4D NifTi data files, using dcm2nii (http://www.mccauslandcenter.sc.edu/mricro/). The BOLD-fMRI data were pre-processed using SPM12 (http://www.fil.ion.ucl.ac.uk/spm). The EPI images were first realigned to adjust for head movements during the image acquisition and the images were corrected for movement-induced distortions (“unwarping”). Data were subsequently inspected for residual movement artifacts, and all movements were <2 mm and the rotations were <2°. Afterwards, the realigned image series were normalized to the stereotaxic space created by Montreal Neurological Institute (MNI), which is defined by an EPI template provided by the SPM12 software package (“Old normalize”). Normalization parameters were estimated from the mean images, generated after the realign and unwarp procedure, and subsequently applied to the realigned time series. The normalized images were resampled with a voxel size of 2 × 2 × 2 mm, and finally smoothed by using a Gaussian kernel of 8 mm.

### General linear model

Following the concept proposed by Di and Biswal (2015), the data were modeled within SPM using a design matrix that consisted only of sinusoidal functions, covering a frequency range 0.005–0.1 Hz. Accordingly, the high-pass filter was set to a liberal cut-off of 360 s. This GLM is only needed for extracting the time course that feed into the sDCM analysis. Hence, only an F-contrast was defined that spanned over all sinusoidal functions.

The dynamic causal modeling approach allows specification of models with up to eight regions. Following the a-prior hypothesis, six anatomically distinct areas were selected for the present analysis covering the core areas of auditory perception, rhythm processing, and reward processing. The respective coordinates of four areas of interest were defined on published coordinates with representative areas for the auditory cortex and the putamen/pallidum. The coordinates for the ventral striatum/nucleus accumbens were identified directly on the MNI template. Since there was no prediction on the hemispheric lateralization, areas were defined for the left and right hemisphere. In total six anatomical areas of interest were defined. These were the auditory cortex (AC) with reference to the subarea Te1 (Morosan et al., 2001), the putamen/pallidum (PA; Grahn and Rowe, 2009) and the ventral striatum/nucleus accumbens (VS), of the left and right hemispheres, respectively (see Table 1 for the exact coordinates). For each region, the time series from the most significant voxel were extracted using a liberal threshold of *p* < 0.05, uncorrected, since the underlying significance of the GLM model fit is of minor relevance for this approach and served only as basis for extracting time courses. If no local maximum was within the range of 8 mm around the target coordinate, the nearest sub-threshold voxel within the 8 mm range was used. This procedure assured that only voxels that showed fluctuations over time were selected for this analysis.

**Table 1:** **The table reports the mean coordinates (±*SD*) for the six areas used in the stochastic DCM analysis**.

**Area**	**X (mm)**	**Y (mm)**	**Z (mm)**
AC-L	−50.6 ± 4.7	−26.7 ± 4.5	0.4 ± 5.2
AC-R	61.2 ± 4.6	−25.5 ± 4.7	5.8 ± 5.1
Pa-L	−29.1 ± 5.5	4.5 ± 4.6	4.2 ± 5.4
Pa-R	25.5 ± 5.7	4.3 ± 6.8	4.2 ± 5.5
VS-L	−11.5 ± 8.6	20.0 ± 4.0	2.3 ± 4.1
VS-R	11.5 ± 4.9	20.2 ± 5.7	0.6 ± 4.5

*AC, Auditory Cortex; Pa, Putamen/Pallidum; VS, Ventral Striatum/Nucleus Accumbens; L, Left, R, Right. The coordinates refer to the MNI space*.

The terms ventral striatum and nucleus accumbens as well as putamen and pallidum are often used interchangeably in the neuroimaging literature. Although these are anatomically and functionally distinct areas, they are often difficult to separate in functional neuroimaging data with low spatial resolution (in the present case 3.4 × 3.4 × 5.5 mm^3^) due their proximities. Therefore, in the following, the terms *ventral striatum*/*nucleus accumbens* as well as *putamen*/*pallidum* will be used to reflect that the precise spatial localization is limited for these areas, given the intrinsic resolution of the raw fMRI data.

### Stochastic DCM

Dynamic causal modeling (DCM) is a method that rests on generative models describing the brain as non-linear, but deterministic system. Those generative models that describe the network of neuronal populations are a set of differential equations that describe how the hidden neuronal states could have generated the observed data. Since the system is described through those equations, the model can be inverted and parameters for the not directly observable hidden states can be estimated (Friston et al., 2011). Originating from task-related fMRI, DCM has been extended to resting-state fMRI, called stochastic DCM (Friston et al., 2011). The stochastic DCM (sDCM) model was defined as a fully connected model, where each node was connected with every other node. The DCM model (DCM12, revision 5729) was defined within SPM12 (rev. 6225). After model estimation, post-hoc Bayesian model optimization was applied (Friston and Penny, 2011). The resulting connectivity estimates were subjected to group-specific one-sample *t*-tests, as well as between-group two-sample *t*-tests using SPSS (ver. 22). A Bonferroni correction was applied, taking into account that 36 *t*-tests were performed. In addition, the hemodynamic response was examined in more detail but analysing the estimated parameter for *transit* (transit time through the “balloon”), *decay* (signal decay), and *epsilon* (neural efficiency). These parameter refer to the revised Balloon model (Friston et al., 2000; Buxton et al., 2004; Buxton, 2012) that express the metabolic response by taking into account the cerebral blood volume, cerebral blood flow, and oxygen extraction rate. A DCM analysis estimates the metabolic parameter *transit* and *decay* for each region separately, while ε is a global parameter per subject.

## Results

**Figure 1:**
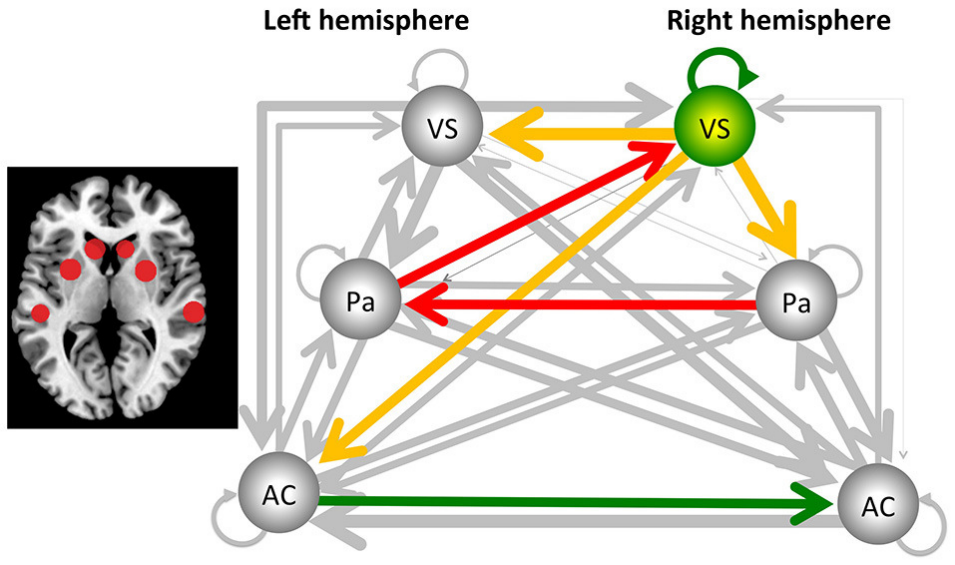
**Results from the stochastic dynamic causal modeling (sDCM) analysis, using the auditory cortex (AC), the putamen/pallidum (PA), and the ventral striatum/nucleus accumbens (VS) of the left and right hemisphere as core areas**. The dark arrows indicate connections that are not significantly different between the music and control groups, and line thickness indicates the strength of the connectivity. Yellow/red arrows indicate a decreased connectivity. In addition, the right ventral striatum/nucleus accumbens reduced its self-inhibition, indicated by the green arrow, as well as changed its hemodynamic response parameter, as indicated by the green sphere. Finally, the green arrow from the left to the right auditory cortex indicates a significant increase of connectivity. The two red arrows indicate that this effect was also significant after Bonferroni correction. The figure to the left indicates the localization of the areas (see Table 1 for the exact coordinates).

The sDCM results revealed significant (Bonferroni-corrected) connections between the auditory cortex, putamen and ventral striatum for both hemispheres and for both groups. More importantly, several significant group differences were detected. In the following, uncorrected and Bonferroni-corrected differences are reported, where the latter ones are marked with “^*^.” All differences are described relative to the control group. Reduced connectivity was detected from the right ventral striatum/nucleus accumbens to the left homolog [*t*_(24)_ = −2.295, *p* < 0.031], to the right putamen/pallidum [*t*_(24)_ = −3.196, *p* < 0.004], and the left auditory cortex [*t*_(24)_ = −2.140, *p* < 0.043]. In addition, reduced connectivity was detected for the connection from the left putamen/pallidum to the right ventral striatum/nucleus accumbens [*t*_(24)_ = −3.901, *p* < 0.001^*^] and from the right to the left putamen/pallidum [*t*_(24)_ = −4.562, *p* < 0.001^*^]. These latter two results were also significant after Bonferroni correction (see Figure 1). Finally, the intrinsic, self-inhibitory connection of the right ventral striatum/nucleus accumbens was reduced in the music group [i.e., less negative, *t*_(24)_ = 2.138, *p* < 0.043]. By contrast, increased connectivity was only detected for the connection from the left to the right auditory cortex [*t*_(24)_ = 2.354, *p* < 0.027].

For the hemodynamic parameter *transit, decay* and *epsilon*, the group comparison demonstrated that the parameter *transit* and *decay* were significantly different for the right ventral striatum/nucleus accumbens, only. Although the corresponding *p*-values do not survive a Bonferroni correction, they are worth being mentioned, since differences were—again—detected only for this area. It appeared that *transit* was significantly reduced [*t*_(24)_ = −2.158, *p* < 0.041], while decay was significantly increased [*t*_(24)_ = 2.202, *p* < 0.038].

## Discussion

With this study, the aim was to investigate brain networks involved in processing a continuously playing piece of rhythmic music. Generally, the analysis of continuous stimulation paradigms is challenging. Therefore, we present for the first time the application of stochastic dynamic causal modeling as a tool for analysing the connectivity in a study with continuous stimulation. The sDCM analysis provided evidence that, when compared to the control group without music stimulation, dance floor-like rhythmic music decreased connectivity from the right putamen/pallidum, via the left putamen/pallidum to the right ventral striatum/nucleus accumbens. Further, the right ventral striatum/nucleus accumbens changed its level of activity by a reduction in self-inhibition and an altered hemodynamic response. In addition, functional connectivity was reduced from the right ventral striatum/nucleus accumbens to the left ventral striatum/nucleus accumbens, the left auditory cortex, as well as the right putamen/pallidum (see Figure 1).

Taking together, the results indicate that mainly the right ventral striatum/nucleus accumbens changed its activity as well as network coupling when listening to this piece of rhythmic, dance-floor-like music. The analysis also shows a significantly reduced connectivity from the right to the left putamen/pallidum, which may reflect a reduced coupling in motor-related brain areas. Future studies may examine further whether this is related to the experience of groove (Stupacher et al., 2013).

The results show that listening to rhythmic music not only changes the level of activation, measured in terms of BOLD signal fluctuation, of the reward system but also causes changes of connectivity within a network that is related to reward processing. The results further support the view that the ventral striatum, i.e., presumably the nucleus accumbens, is deeply involved in processing music-evoked emotions and reduces its connectivity to other areas. This does not mean a reduced activation, but rather acting on a different time scale (Mueller et al., 2015). In fact, the significantly reduced self-inhibition as well as the changed hemodynamic parameter of the right ventral striatum/nucleus accumbens indicate an increased level of activation. The results are also in line with a recent meta-analysis by Koelsch (2014), who referred to the right ventral striatum and in particular to the right nucleus accumbens as an important structure for processing music-induced emotions. In this respect, the nucleus accumbens, which is part of the dopaminergic system, is seen as part of the reward processing system which is mainly active during experiencing reward, and its activity may correlate with the reward value (Salimpoor et al., 2013, 2015; Ikemoto et al., 2015; Zatorre, 2015). This is true for any type of reward, in particular biologically relevant rewards such as food or sex, but also money (Daniel and Pollmann, 2014), and even fairness (Cappelen et al., 2014). By contrast, the dorsal striatum/caudate is generally more related to anticipation of reward but also to music-induced frisson (Koelsch et al., 2015; Zatorre, 2015). This notion is further supported by a PET study that indicated increase of dopamine release that was different for the ventral and dorsal striatum. The dorsal striatum/caudate appeared to be more involved in the anticipation and prediction of reward, while the ventral striatum/nucleus accumbens demonstrated the strongest dopamine release while experiencing an emotional response to music (Salimpoor et al., 2011).

The results presented here may contradict an fMRI study by Salimpoor and colleagues, who detected an increased connectivity between the auditory cortex and the ventral striatum (Salimpoor et al., 2011). However, one has to bear in mind that the present study used a continuous stimulation paradigm with an unfamiliar piece of music that lasted for 10.16 min and thus much longer than the stimuli of most other studies that typically last less than a minute. Further, all measures are derived from measures of BOLD fluctuations and how they propagate through the specified network. In this respect, results may not be directly comparable to studies, where connectivity measures were based on task contrasts.

Finally, it should be mentioned that the sDCM analysis also indicated an increased connectivity from the left to the right auditory cortex when compared to ordinary resting-state fMRI. This confirms the validity of our approach of analysing data from a continuous stimulation paradigm with sDCM. Moreover, an increased connectivity from left to right was reasonable to expect, since the right auditory cortex is assumed to be more dominant during the processing of tonal information, like music (Tramo, 2001; Zatorre et al., 2007).

The results presented here are also significant from a methodological perspective. The unconstrained DCM model not only confirmed the notion about the right ventral striatum, but it also confirmed the validity of the presented sDCM approach. Note that for the first time a continuous stimulation protocol was combined with a stochastic DCM approach. A stochastic DCM model is set up as a fully connected model without any constraints. Therefore, it is a significant result that the contribution of the right ventral striatum evolved naturally out of the model estimation. Further, there are no significant group differences of the hemodynamic parameter or of the self-inhibition for the auditory system. This may indicate that continuous auditory stimulation does not change hemodynamic fluctuations within the auditory system, when compared to resting-state fMRI, although the level of activation might be different.

However, there are important limitations to the selected approach that one also has to bear in mind and that restrict the general conclusions that can be drawn from the presented results. Firstly, due to methodological constrains, the present study only compares music perception to resting state and used only one piece of music. Although there is strong evidence that the detected effects are mostly related to the presentation of rhythmic music and that the majority of the participants experienced this music as pleasant, one cannot entirely rule out that comparable effects may have emerged when listening to other types of (pleasant) stimuli in a similar passive and “resting” situation. In addition, the experience of groove wasn't formally assessed in this study although several participants of the music group reported this, but one might also want to speculate whether the observed decoupling could be related to suppressing the urge to move along. Secondly, the DCM approach generally limits the number of areas that can be included in a model, and these areas should have a certain distance to avoid overlapping effects through smoothing. Accordingly, the selection of the areas of interest was based on the a-prior hypothesis described in the introduction that comprised the auditory cortex, the basal ganglia and the reward system. Therefore, only these six anatomically distinct areas were included in the model, but future study may include additional areas like the ventromedial prefrontal cortex or amygdala. Finally, fMRI studies with 13 participants per group are still within the standard range, but larger sample sizes are required and recommended for future studies for drawing more general conclusions.

## Conclusion

This study demonstrated a reduced functional connectivity and thus less constrained activation of the reward processing system, namely the right ventral striatum and nucleus accumbens, while listening to a several minutes-long pleasurable and rhythmic piece of music—as compared to silence. The results give a deeper insight into the power of music and show how easily and how strongly rhythmic music interacts with the affect and basal ganglia system, and that emotional experiences during listening to music may be generated through a stronger activation and, simultaneously, reduced functional connectivity of the reward system. Thus, “*The powers of cognition that are set into play by this representation [of a beautiful object] are hereby in a free play, since no determinate concept restricts them to a particular cognition*” *(Kant, 1790)* (Guyer, 2002).

## Author contributions

HB contributed to the analysis of the data and writing of the article. BO contributed to the planning, stimulus preparation, and writing of the article. KS contributed to the planning, stimulus preparation, performance, analysis, and writing of the article.

### Conflict of interest statement

The authors declare that the research was conducted in the absence of any commercial or financial relationships that could be construed as a potential conflict of interest.
